# Quantifying Cardiac, Respiratory, and Low Frequency Components of CSF Motion From fMRI Inflow Effects

**DOI:** 10.1002/mrm.70438

**Published:** 2026-05-17

**Authors:** Pontus Söderström, Cecilia Björnfot, Britt M. Andersson, Jan Malm, Anders Eklund, Anders Wåhlin

**Affiliations:** ^1^ Department of Applied Physics and Electronics Umeå University Umeå Sweden; ^2^ Department of Diagnostics and Intervention, Biomedical Engineering and Radiation Physics Umeå University Umeå Sweden; ^3^ Umeå Center for Functional Brain Imaging (UFBI), Umeå University Umeå Sweden; ^4^ Department of Clinical Science, Neurosciences Umeå University Umeå Sweden

**Keywords:** cerebrospinal fluid flow, glymphatic system, inflow effect, quantitative flow assessment, resting‐state fMRI

## Abstract

**Purpose:**

Cerebrospinal fluid (CSF) flow oscillations have emerged as a potentially important marker related to brain clearance, but their acquisition often relies on specialized imaging MRI sequences. The purpose of this work was to enable quantitative assessment of CSF flow associated with cardiac, respiratory, and low‐frequency cycles using widely available functional magnetic resonance imaging (fMRI) acquisitions.

**Methods:**

A method was developed to translate fMRI‐derived CSF inflow signals into quantitative flow rates. This approach modeled the spin‐history of an oscillating ensemble of molecules. Validation was performed using phantom experiments with cardiac‐, respiratory‐, and low‐frequency‐like oscillatory flow. The method was further applied to resting‐state data from 48 older adults (68–82 years, 19 women) to characterize CSF flow at the foramen magnum.

**Results:**

Phantom experiments demonstrated excellent correlations between estimated and true velocities for cardiac‐ and respiratory‐like frequencies (*r* = 0.94 and 0.97, respectively) and moderate correlation for the low‐frequency‐like oscillation (*r* = 0.58). In the population cohort, median CSF stroke volumes were 0.77 [0.57, 1.09] mL for the cardiac cycle, 0.38 [0.26, 0.88] mL for the respiratory cycle, and 0.26 [0.14, 0.39] mL for the low‐frequency cycle.

**Conclusion:**

The proposed spin‐history modeling method enabled quantitative estimation of CSF flow components using a conventional fMRI dataset and showed that the cardiac cycle dominates CSF motion at the foramen magnum.

## 1 Introduction

Cerebrospinal fluid (CSF) flow oscillations occur in response to cardiac, respiratory, and low frequency vascular oscillations [1, 2]. Measuring this compensatory response can provide important information regarding intracranial compliance [3, 4]. Furthermore, CSF pulsation measurements may be informative of the physiological driving forces that can support brain clearance [5]. Brain clearance, as currently understood, is performed by the glymphatic system [6, 7] where CSF is suggested to follow perivascular spaces, driven by physiological mechanisms such as cardiac pulsations [8, 9], respiratory‐dependent pressure changes [10, 11], as well as slower vascular modulations of neural origin [12, 13]. These physiological drivers can be studied in macroscopic CSF regions such as the fourth ventricle and the foramen magnum [14, 15], and while CSF flow in the subarachnoid space is not the same as glymphatic flow, it is still believed to be related [16, 17]. In this context, CSF flow at the foramen magnum may be especially informative since it, to a greater extent, reflects the total volume of CSF displacement occurring in response to cerebrovascular volume changes, as suggested by the Monro‐Kellie doctrine [18], compared with measurements of CSF flow at the fourth ventricle.

Functional magnetic resonance imaging (fMRI) is a widely used technique to measure neural activity non‐invasively, primarily through the detection of blood oxygen level‐dependent (BOLD) signals, and large databases from studies focusing on cognition in aging and dementia are already established [19, 20, 21]. While the main purpose of fMRI is to detect brain activations through local changes in blood oxygenation, signal fluctuations related to CSF flow are visible at edge slices. Importantly, in whole brain fMRI, the lower edge‐slice typically contains CSF surrounding the spinal cord. The fMRI signal increases when unsaturated CSF enters the imaging volume, a mechanism called the inflow effect [22]. By exploiting the inflow effect, researchers have indirectly measured CSF movement through signal intensity fluctuations [14]. These observations have revealed that the change in global BOLD signal is coupled with the CSF flow during sleep, suggesting that CSF dynamics is linked to neural activity while sleeping. Furthermore, a significant coupling between global BOLD and CSF signal changes has also been seen during resting‐state fMRI (as typically acquired in the previously mentioned large datasets), where a reduced correlation was associated with neurological disorders such as Alzheimer's [15] and Parkinson's disease [16]. These examples, however, have isolated slower frequencies in the CSF flow spectrum, and a quantitative description regarding the importance of slow waves, respiration and the cardiac cycle to CSF motion is lacking.

Importantly, because of the oscillating nature of CSF flow, the inflow signal is not a direct reflection of instantaneous velocity, but rather the proportion of the fluid that has been replaced since last excitation and the spin‐history. The complex dependency on spin‐history adds to the challenge of translating the inflow effect to velocity, potentially obscuring true relationships between the physiological cycles and CSF oscillations. This issue becomes of particular significance when the sampling frequency is comparable to or lower than twice the heart rate. Under such conditions, typical for large‐scale fMRI data collections, the inflow signal of cardiac‐related flow will be aliased and falsely appear as a low frequency modulation, as the Nyquist criterion is not met [23]. While previous studies have utilized analytical models to estimate flow rates [24, 25, 26], these require assumptions such as unidirectional flow that are better suited for blood flow than CSF flow. Developing a robust method to quantify CSF flow from conventional fMRI acquisitions could open new opportunities to investigate the relationship between CSF flow and various cognition‐ and aging‐related metrics that are commonly acquired in ongoing fMRI studies. Such relationships have the potential to provide valuable insights into areas including brain clearance and the progression of neurodegenerative diseases [27].

Here we propose a method that takes spin history into account to quantify CSF flow from the observed inflow effect in fMRI data. The method does not depend on rapid sampling and can be implemented with standard fMRI acquisitions. Conceptually, this approach can be understood as a forward‐model inversion of spin‐history under oscillatory flow. We validated the method against phantom measurements and applied it to a typical fMRI dataset to quantify the magnitude of cardiac, respiratory, and low frequency cycles for CSF flow at the level of foramen magnum, thus directly addressing the question regarding the importance of different physiological cycles to CSF flow.

## 2 Methods

### 2.1 Subjects

Sixty‐one elderly individuals were randomly selected from the population registry in Umeå, Sweden [28]. Eight did not complete the fMRI and five were excluded due to poor physiological recordings during the scan (pulse oximeter and respiratory bellows). Thus, the study population consisted of 48 individuals (68–82 years, 19 women), with characteristics summarized in Table 1. Prior to the MRI scans, all participants underwent a medical examination, during which blood pressure was measured in a seated position on the left arm. Relevant medical conditions and medications were also noted. Cognition was tested in all participants using a Montreal Cognitive Assessment (MoCA) test.

**TABLE 1:** Descriptive characteristics of the participants in this study (*N* = 48). Categorical variables are given as *N* (%), whereas continuous variables are presented with mean ± SD.

Age (Years)	76.1 ± 4.0
Sex (Men/Women)	29/19 (60/40)
BMI (kg/m^2^)	24.9 ± 3.5
SBP (mmHg)	135 ± 16
DBP (mmHg)	75 ± 10
MoCA score	26.2 ± 2.4
Diabetes mellitus, *N* (%)	6 (13)
Hypertension, *N* (%)	28 (58)
Hyperlipidemia, *N* (%)	25 (52)
Atrial fibrillation, *N* (%)	10 (21)
Transient ischemic attack, *N* (%)	3 (6)

Abbreviations: BMI, body mass index; DBP, diastolic blood pressure; MoCA, montreal cognitive assessment; *N*, number of participants; SBP, systolic blood pressure; SD, standard deviation.

The study was conducted according to the ethical principles outlined in the Helsinki Declaration, and the Swedish Ethical Review Authority has approved the study (approval: 2020‐03710). Informed consent was obtained both in writing and orally from all participants. To only include participants who could provide informed consent, a minimum MoCA score of 20 out of 30 possible points was required for participation.

### 2.2 MRI Protocol

MRI acquisitions including resting‐state fMRI and high‐resolution T_1_‐weighted volumes were acquired using a 3 T scanner (Discovery MR 750; GE Healthcare, Milwaukee, Wisconsin), with a 32‐channel head coil. Resting‐state fMRI was obtained through a BOLD‐contrast sensitive T_2_*‐weighted single‐shot gradient echo‐planar imaging (EPI) sequence. The imaging volumes were collected with 37 axial slices in an interleaved order with a slice thickness of 3.4 mm and 0.5 mm spacing. The imaging parameters were set as follows: TR/TE 2000/30 ms, flip angle 80°, and field of view (FOV) 25 × 25 cm^2^ with in‐plane resolution 128 × 128 voxels. The sequence began with 10 dummy scans so that the magnetization had reached a steady state before any data was collected. Furthermore, physiological recordings including pulse and respiration were collected throughout the scanning period (6 min) using a peripheral pulse unit and respiratory bellows.

A baseline anatomical inversion recovery prepared T_1_‐weighted volume was collected using a fast spoiled gradient echo (FSPGR) sequence with the following imaging parameters: TI/TR/TE 450/8.2/3.2 ms, flip angle 12°, 1 mm slice thickness, in‐plane resolution 0.93 mm, FOV 25 × 25 cm^2^ with 176 axial slices, and parallel imaging factor of 2. Variable flip angle T_1_‐mapping [29] was implemented with FSPGR acquisitions and flip angles of 2° and 12° using the same FOV and resolution as the anatomical scan. From these images, subarachnoid space CSF T_1_‐times for each participant were extracted by using an intersection mask based on a baseline T_1_‐value cutoff of 4 s and a FreeSurfer classification as CSF.

The MRI acquisitions were conducted within a broader quantitative imaging protocol that included intravenous gadolinium injection [28], which was not utilized in the present study. In this protocol, the baseline T_1_‐weighted volumes were collected before the injection of gadolinium (Dotarem), whereas the resting‐state fMRI acquisition was approximately 3 h after contrast injection. Gadolinium potentially impacts the BOLD signal, but it has been reported that even when acquisition occurs close to the injection time, no statistically significant mean signal change is observed [30]. Moreover, while intravenous injections of gadolinium can have a measurable impact on the T_1_‐times of CSF [31] our measurement protocol involved measuring the average T_1_‐times before and after the fMRI acquisition. Therefore, the effect of gadolinium on the T_1_‐values used in the model was accounted for.

### 2.3 fMRI Processing

**FIGURE 1:**
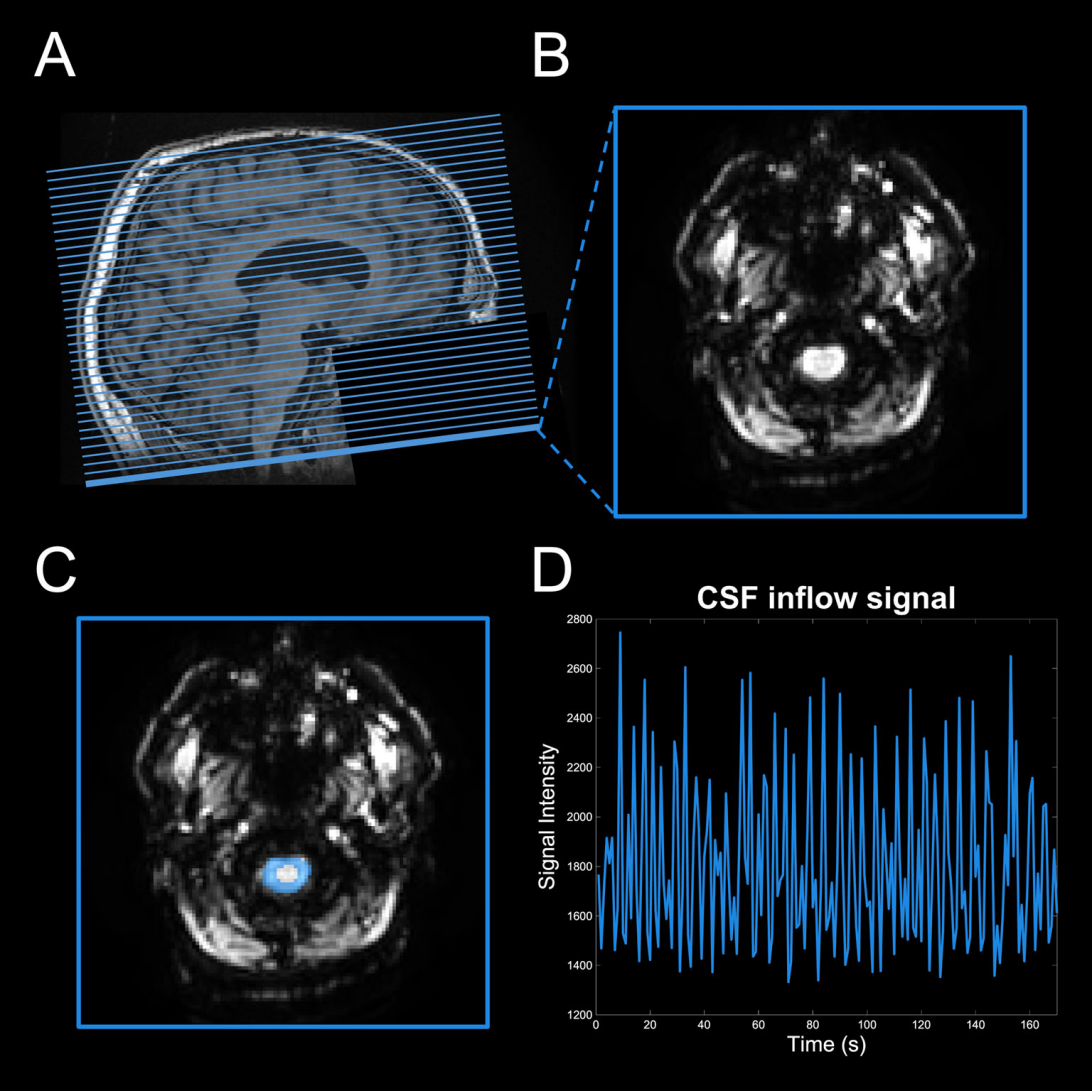
Illustrations of the CSF signal extraction in fMRI. (A) T_1_‐weighted image showing fMRI slice coverage. (B) The bottom fMRI slice with the CSF region of interest highlighted in (C). This region was used to extract the inflow effect time series shown in (D).

Before any preprocessing, we manually drew regions of interest (ROIs) in the area of foramen magnum on the bottom slice of the fMRI volume to capture the inflow effect of CSF. The manual drawing was based on the difference between each voxel's maximum and minimum signal intensity, thereby highlighting voxels with significant movement. The ROIs were divided into two classes based on K‐means clustering on the EPI signal to separate CSF voxels from non‐CSF voxels, where the visually identified CSF component consistently expressed higher area fraction, consistent with the ROI construction. The voxel division was visually inspected to verify a representative segmentation of the CSF region and further details in the class separations, as analyzed with principal component analyses as well as the Silhuette method, are seen in Figure S1. The signal related to CSF movement was then calculated as a spatial average of all CSF voxels, and the resulting time‐series was normalized by division of its mean. For the BOLD signal analysis, realignment, slice timing, and co‐registration to a T_1_‐weighted volume was performed on each fMRI session using SPM12 [32]. A whole brain cortical gray matter ROI was created using cortical surface segmentations in FreeSurfer 6.0 [33] and removal of physiological noise was performed using RETROICOR [34]. After that, the global BOLD signal in gray matter was extracted from the mean value of all voxels within the gray matter ROI after voxel‐wise normalization to z‐score. The signal was then detrended using a polynomial of degree 2 prior to the application of a band‐pass filter (0.01–0.1 Hz), where the processed signal serves to characterize *low frequency* throughout the study. The process of extracting the EPI signal associated with CSF movement from the bottom slice is illustrated in Figure 1. Since high displacements impact the spin history, the framewise displacement was calculated to exclude participants with high movement during the fMRI scan. Here, we excluded participants with a mean framewise displacement greater than 0.5 mm or a maximum framewise displacement greater than 3 mm (*N* = 5) as motion induced changes in spin history are not considered in the model and may therefore have a negative impact on the fit of the simulated signals.

### 2.4 Modeling CSF Dynamics

**FIGURE 2:**
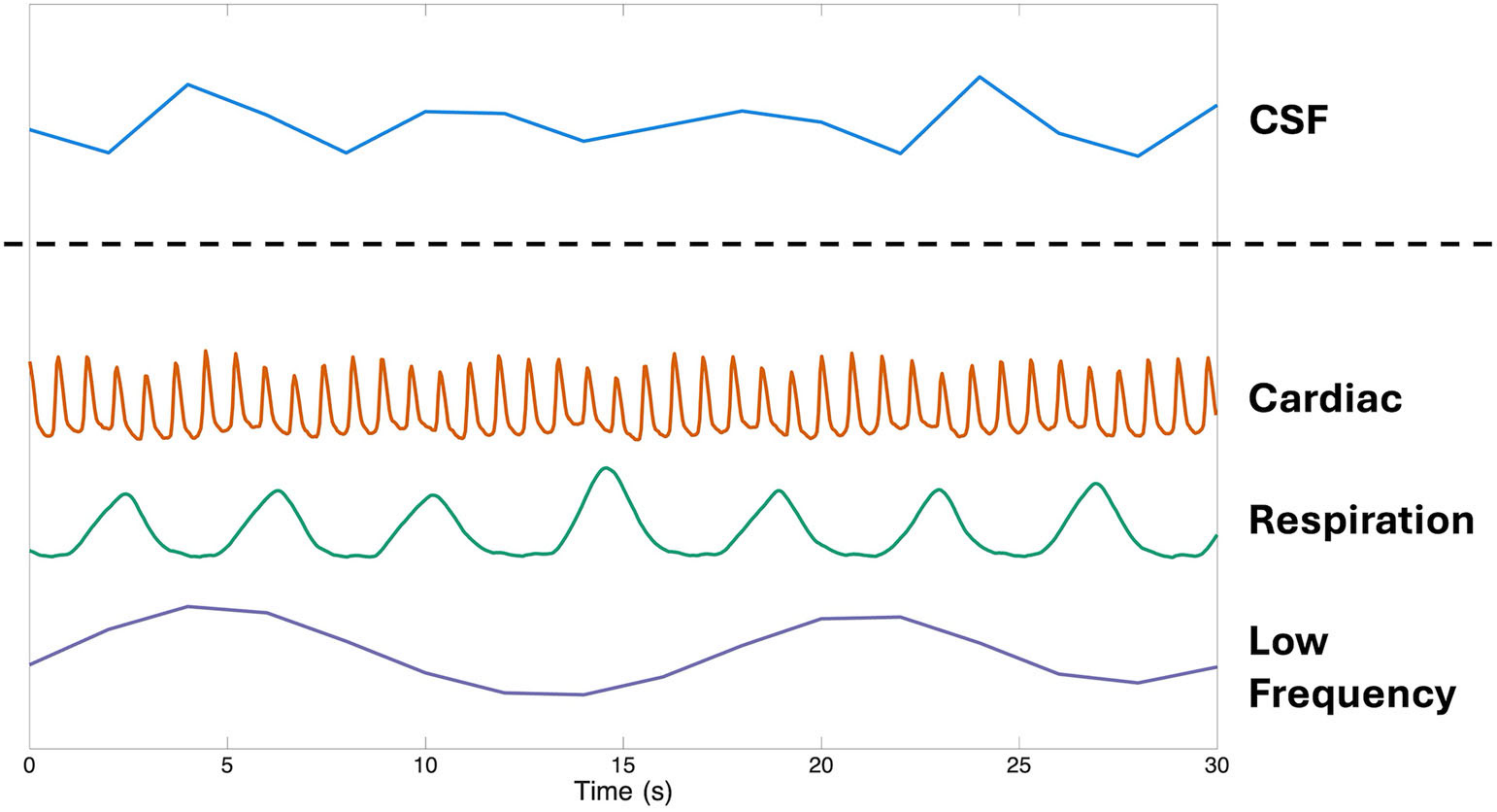
Time‐series of physiological signals extracted during the fMRI acquisition. The CSF signal was derived from EPI signal fluctuations within the bottom‐slice CSF region of interest (ROI). Cardiac and respiratory signals were recorded via pulse oximetry and respiratory bellows, respectively, while low frequency oscillations were estimated from the global BOLD signal. Note that the sampling frequency of the CSF signal fluctuations is that of the fMRI sequence (i.e., 2 s).

The CSF dynamics modeling presented in this study is based on the Monro‐Kellie doctrine [18], where the total volume of brain, intracranial blood and CSF is assumed to be constant. This suggests that CSF buffers variations in blood volume by flowing in and out of the spinal compartment, which we want to quantify from the EPI signal fluctuations around foramen magnum. The intracranial blood volume is influenced by cardiac pulsations, respiratory‐driven pressure changes, and slower fluctuations related to neural activity and autoregulatory mechanisms (vasomotion). Therefore, we constructed a model to mathematically describe the signal fluctuations caused by CSF movement, where the CSF velocity is also assumed to depend on cardiac pulsations, respiratory variations, and low frequency oscillations derived from the global BOLD signal. The physiological signals used in the modeling are visualized together in Figure 2. We assumed that the cardiac, respiratory, and low frequency components of the CSF velocities are quasi‐periodic, captured by the phases calculated from recordings of pulse, respiration, and the global BOLD signal.

The phases are calculated with a previously described method that uses the Hilbert‐Transform (HT) of a physiological signal to extract the corresponding phases [35]. This approach was selected specifically because CSF oscillations are neither purely sinusoidal nor strictly stationary. The HT approach allows for the extraction of instantaneous phase information despite the natural irregularity and non‐sinusoidal morphology of real physiological waves [36]. With this method, the cardiac phase ($\varphi_{C}$) was calculated via the HT of the pulse oximetry data. Similarly, the respiratory phase ($\varphi_{R}$) was calculated through the HT of the respiratory bellows data. Lastly, for the low frequency component, we used the time series representing the negative derivative of the global BOLD signal to calculate the phase ($\varphi_{\mathrm{SV}}$) via the HT, since this derivative should represent the rate of change in cerebral blood volume [14]. Using the assumptions above, the CSF velocity over time (t) was written as,

(1) $$\begin{array}{ll} v_{\mathrm{CSF}}\left(t\right) & =v_{C}\cos\left(\varphi_{C}\left(t\right)+\delta_{C}\right)+v_{R}\cos\left(\varphi_{R}\left(t\right)+\delta_{R}\right)\\ & \;+v_{\mathrm{SV}}\cos\left(\varphi_{\mathrm{SV}}\left(t\right)+\delta_{\mathrm{SV}}\right), \end{array}$$

where $v_{C}$, $v_{R}$, and $v_{\mathrm{SV}}$ represent the magnitude of the cardiac, respiratory and low frequency components of the CSF velocity. Moreover, $\delta_{C}$, $\delta_{R}$ and $\delta_{\mathrm{SV}}$ are phase shifts between the CSF velocity and the cardiac, respiratory and low frequency phases respectively. To avoid the ambiguity of selecting specific integration windows, CSF displacement was derived as a continuous, time‐dependent signal. This was achieved by integrating $v_{\mathrm{CSF}}$ continuously over time using a quasi‐stationary approximation. For a velocity amplitude $v_{0}$ and instantaneous phase φ(t), the following approximation (derived via integration by parts) holds: $\int_{0}^{t}v_{0}\cos\left(\varphi \left(\tau \right)\right)d\tau \approx \frac{v_{0}}{\varphi^{\prime }\left(t\right)}\sin\left(\varphi \left(t\right)\right)$, which relies on the assumption that $\varphi^{\prime \prime }\left(t\right)\ll {\left(\varphi^{\prime }\left(t\right)\right)}^{2}$. A least squares estimation of the velocities and phase shifts for each velocity component was acquired by,

(2) $$v,\delta =\arg\;\min_{v,\delta }{\left\|S-\tilde{S}\left(v_{\mathrm{CSF}}\right)\right\|}_{2}^{2},$$

where S is the measured inflow signal, $\tilde{S}$ is the simulated signal, $v=\left\{v_{C},v_{R},v_{\mathrm{SV}}\right\}$, and $\delta =\left\{\delta_{C},\delta_{R},\delta_{\mathrm{SV}}\right\}$. The optimization problem was minimized using particle swarm [37] with upper and lower bounds on the velocities corresponding to ±50 mm/s for each component.

### 2.5 Simulations of CSF Inflow Signal

**FIGURE 3:**
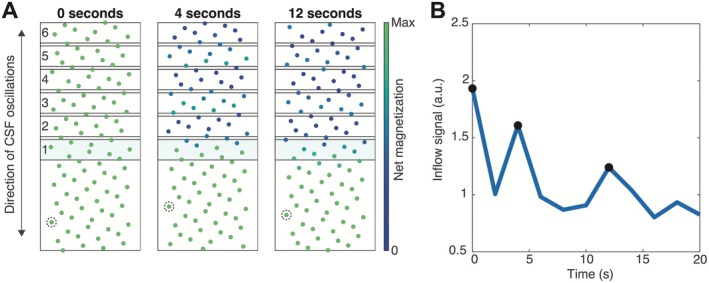
Visualization of the simulation approach. (A) Magnetizations of elements in an ensemble that oscillates in a direction perpendicularly to the imaging slices. Magnetization is provided at three example time points where the edge slice is being excited (illustrated by the light blue color). To help visualize the motion, one element has been highlighted by a dashed line. (B) The signal in the edge slice, with the example time points in (A) marked.

An illustration of the simulation is provided in Figure 3, as well as in Video S1. The simulations began with setting *N* equispaced elements (i.e., the oscillating ensemble) in a region around the bottom slice of the imaging volume. Each of the elements were assigned an initial magnetization $M_{0}$ along the main magnetic field, representing elements of CSF. The geometry of the cervical spinal canal (extending from the foramen magnum) was approximated as a uniform, straight tube in the modeling. The position of each element was updated according to $v_{\mathrm{CSF}}\left(t\right)$, where a plug flow was used as flow profile of the CSF flow. The inflow effect was simulated by continuously updating the magnetization of moving CSF throughout the fMRI sequence, based on solutions to the Bloch equations [38] and by considering excitation of multiple slices. Thus, protocol‐specific parameters are supplied to the optimization code in order to make representative simulations (www.github.com/Buntess/fMRI‐Flow‐Quantification). With the main magnetic field along the *z*‐direction, the initial unperturbed net magnetization is along the z‐direction with magnitude $M_{0}$. Directly after the application of a radiofrequency (RF) pulse of flip angle θ, the magnetization parallel to the main magnetic field ($M_{z}$) and the transverse magnetization ($M_{⊥}$) become

(3) $$\begin{array}{c} M_{z}^{\left(1\right)}\left(0\right)=M_{0}\cos\left(\theta \right),\\ M_{⊥}^{\left(1\right)}\left(0\right)=M_{0}\sin\left(\theta \right), \end{array}$$

where the superscript indicates the number of RF pulses applied to a specific element. From the Bloch equations, the relaxation of the magnetizations at time t after the ith RF pulse become

(4) $$\begin{array}{cc} M_{z}^{\left(i\right)}\left(t\right) & =M_{0}\left(1-e^{-t/T_{1}}\right)+M_{z}^{\left(i\right)}\left(0\right)e^{-t/T_{1}},\\ M_{⊥}^{\left(i\right)}\left(t\right) & =M_{⊥}^{\left(i\right)}\left(0\right)e^{-t/T_{2}}, \end{array}$$

where $T_{1}$ and $T_{2}$ are the T_1_ and T_2_ relaxation times. A general expression for the parallel and transverse magnetization after *i* RF pulses (assuming complete spoiling between RF pulses) is

(5) $$\begin{array}{c} M_{z}^{\left(i\right)}\left(0\right)=M_{z}^{\left(i-1\right)}\left(∆T_{i}\right)\cos\left(\theta \right),\\ M_{⊥}^{\left(i\right)}\left(0\right)=M_{z}^{\left(i-1\right)}\left(∆T_{i}\right)\sin\left(\theta \right), \end{array}$$

where $∆T_{i}$ is the time between the two succeeding pulses experienced by an element and will be equal to TR only if both excitations occur within the same imaging slice. However, due to CSF motion, the element may undergo excitations in other slices, resulting in $∆T_{i}$ differing from TR.

The magnetizations of the elements were updated according to the Equations ((3), (4), (5)) together with the settings of the fMRI sequence, which includes the excitation of multiple slices. Physiologically, within the CSF space, there is some degree of dispersion that would cause blurring of the magnetization between adjacent positions. To account for this, the spin ensemble was effectively treated as a continuous density distribution, and the magnetization at each time step and position was convolved with a Gaussian kernel. This procedure is mathematically equivalent to simulating the physical spread of individual elements via dispersion in the limit of infinitesimal time steps. The modeling utilized a dispersion coefficient of 6 cm^2^/min, which was chosen based on phantom validation and is of the same order of magnitude as the previously reported dispersion coefficient of spinal CSF [39]. The T_1_‐value for CSF was set to the cohort average 4738 ± 186 ms, whereas the T_2_‐value was set 1600 ms based on the literature [40]. The signal at the bottom slice was acquired by the mean transverse magnetization of all elements localized within the slice, evaluated at TE for each excitation of the bottom slice. Moreover, the simulated signal was normalized by its mean, eliminating the need to determine $M_{0}$.

### 2.6 Model Validation

The effects of overfitting on the model were examined by fitting the model when interchanging each participant's cardiac, respiratory, and low‐frequency recordings by another participant's recordings. For each participant, the physiological recordings were interchanged with a participant that displayed a low correlation of the physiological recordings, using a metric that was calculated as the maximum absolute cross‐correlation between each of the three physiological signals. However, evaluating participants independently to find low‐correlation pairs could lead to overlapping assignments and potentially introduce bias if any participant's gating is used multiple times. Therefore, the Munkres algorithm [41] was applied to globally minimize the correlation between original and swapped recordings while ensuring a one‐to‐one reassignment where every gating is used exactly once. The explained variance (R^2^) from model fits using swapped recordings was presented with those derived from the true recordings to compare the model's ability to describe the EPI‐inflow signal when using both true and false gating data. Furthermore, given that the model depends on the selection of the T_1_ relaxation time, we examined the effect of this parameter by comparing CSF stroke volumes obtained when the group average T_1_‐time (4738 ms) was used compared to the individually calculated T_1_‐times. Moreover, we tested how well the model predicts low‐frequency CSF stroke volumes without cardiac or respiratory gating. To do this, we compared results from a simplified model (fitting only the low‐frequency component) to those from the full model.

The model was further validated using a phantom mimicking oscillatory flow of CSF. Here a hollow cylinder with a length of 9.2 cm and inner/outer diameter of 2.0/4.3 cm was cast in agar (30 g/L) to avoid water/plastic interfaces. An in house developed syringe pump was used to generate oscillatory flow inside the hollow cylinder, which followed a sine‐curve with a fixed oscillatory frequency (see Figure S2 for schematic illustration of the setup). The frequency of the oscillatory flow was set to 0.93 Hz, 0.23 Hz, and 0.077 Hz respectively to mimic cardiac, respiratory, and low frequency cycles. Phantom flow accuracy was validated by comparing the prescribed pump volumes against the distal passive syringe displacement measured via video recordings (*N* = 6, 0.575–1.105 mL). Distal volume displacements were within 5% of the prescribed volumes (*r* = 0.99), confirming excellent flow fidelity. This validation was executed at the cardiac frequency, where the risk of compliance‐induced volume dampening is inherently highest. For the phantom measurements, we used water as flowing medium, and the imaging parameters of the fMRI acquisitions were the same as the in vivo scan except for the scanning time that was now decreased to 2 min. The evaluation was performed with 6 different velocity amplitudes for each of the three frequencies, with cardiac velocities ranging between 0 and 20 mm/s, respiratory within 0 and 5 mm/s, and low frequency within 0 and 2.5 mm/s. The simulations of the inflow signal for the oscillating flow in the phantom were repeatedly run with different dispersion coefficients to evaluate the robustness of the model.

In addition to the physical validation, a digital phantom was developed to extensively test the model and its underlying assumptions. Multiple experiments were conducted within this computational framework. To evaluate element density requirements, simulations were performed using varying spatial resolutions ranging from 1 to 100 elements per millimeter. The model's sensitivity to noise was also assessed by simulating signals using physiological parameter ranges and subsequently adding random Gaussian noise derived from in vivo fit residuals. Finally, the validity of the straight‐pipe approximation when real anatomy may express a change in CSF area in the longitudinal direction of flow was tested by generating signals within a funnel geometry (expanding from a base area of 125 mm^2^ to 440 mm^2^ over a 10 mm length) and fitting these signals with the standard constant‐area model. Moreover, the assumption that a plug flow is representative of the CSF flow profile was validated by simulating signal variations with different flow profiles and fitting a plug flow to the corresponding signal change. Here we simulated signals corresponding to flow profiles of a laminar flow inside a cylinder, a laminar flow inside an annulus with outer radius 3 times larger than the inner radius, and a plug flow. The simulations were repeatedly run with mean velocities ranging from 1 mm/s to 25 mm/s, and the fitted plug velocities were compared to the mean velocities of the different flow profiles.

### 2.7 Statistics

All statistical analyses were conducted in MATLAB (version 9.13.0. Natick, Massachusetts: The MathWorks Inc.). Normality of the reported variables was assessed using skewness and kurtosis by calculating *z*‐scores, with values within ±1.96 indicating normality. Normal variables are reported as mean ± standard deviation, whereas non‐normal variables are reported with median and interquartile range. Pearson correlation coefficient was used to measure associations between two variables and *p* values were considered significant at the 0.05 level.

## 3 Results

### 3.1 Phantom Validation

**FIGURE 4:**
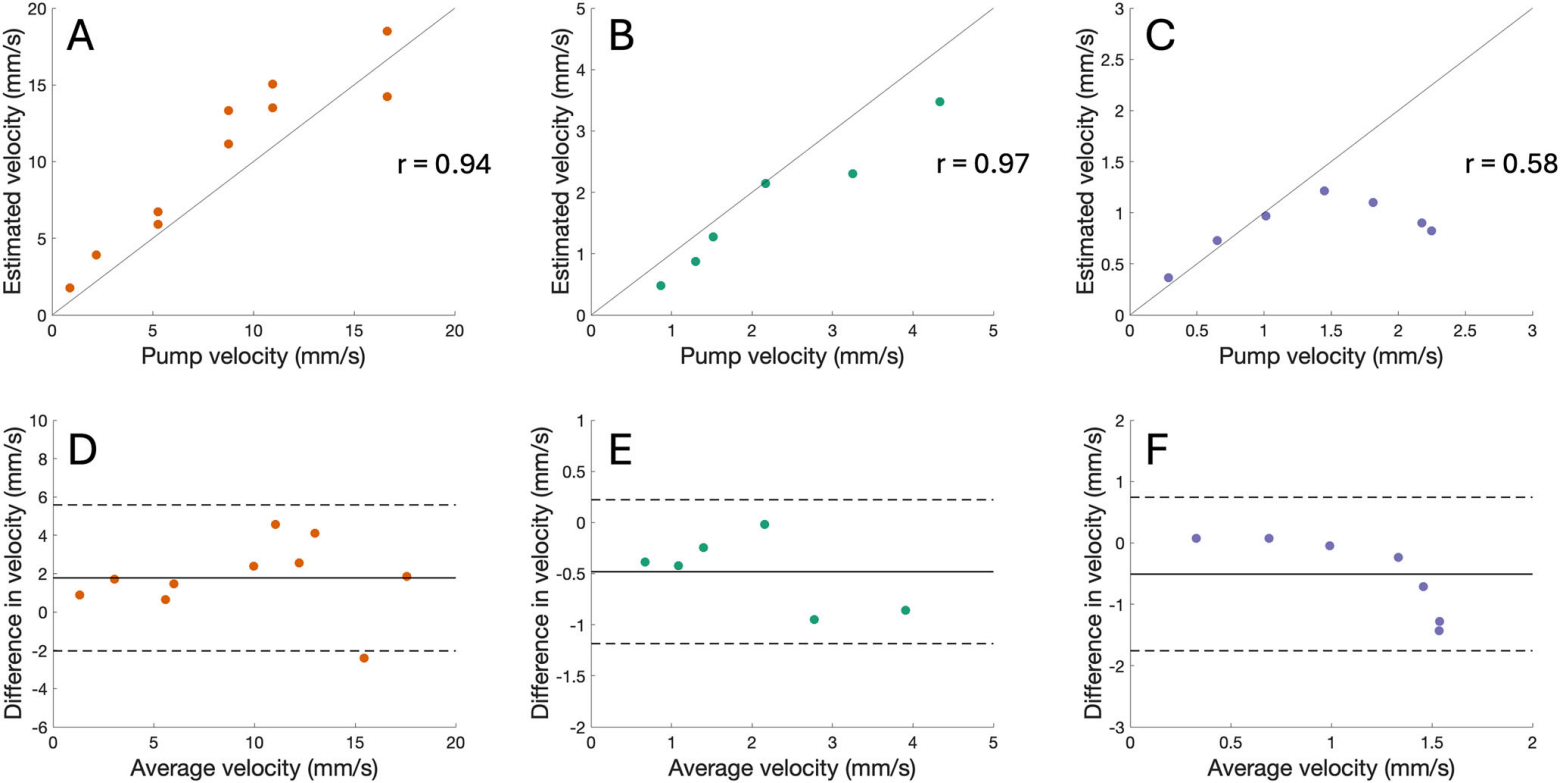
Results from phantom measurements when using a dispersion coefficient of 6.00 cm^2^/min. (A) Estimated velocities vs. pump velocities for cardiac reflected frequency (0.93 Hz). (B) Estimated velocities vs. pump velocities for respiratory reflected frequency (0.23 Hz). (C) Estimated velocities vs. pump velocities for low frequency reflected frequency (0.077 Hz). (D) Bland–Altman analysis of estimated vs. pump velocities for the cardiac frequency. (E) Bland–Altman analysis for the respiratory frequency. (F) Bland–Altman analysis for the low frequency.

The correlation coefficients and linear fits between estimated velocity and the applied pump velocity for all simulations are summarized in Table 2, with all the correlations being statistically significant. Because the low frequency simulations exhibited a strong linear relationship with velocities up to 1.5 mm/s, but a weaker relationship at higher velocities, we included a separate column in Table 2 presenting data limited to this velocity range. The estimated velocities for each of the three frequencies together with the applied pump velocity are displayed in Figure 4 for the simulations with a dispersion coefficient of 6.00 cm^2^/min.

**TABLE 2:** Results from phantom measurements when using different values of the dispersion coefficient.

	Cardiac (0.93 Hz)			Respiratory (0.23 Hz)			Low frequency (0.077 Hz)			Low frequency (0.077 Hz) (Velocities ≤ 1.5 mm/s)		
Dispersion coefficient (cm^2^/min)	*r*	*α*	*β*	*r*	*α*	*β*	*r*	*α*	*β*	*r*	*α*	*β*
2.00	0.9270	1.7316	0.6500	0.9641	0.0148	0.5592	0.5735	0.4308	0.1530	0.9899	0.1547	0.5279
3.00	0.9277	2.0623	0.7445	0.9729	−0.0573	0.6689	0.5685	0.4820	0.1742	0.9835	0.1698	0.5966
4.00	0.9281	2.1183	0.8338	0.9670	−0.0097	0.6959	0.5651	0.5265	0.1842	0.9862	0.1876	0.6446
5.00	0.9214	2.3760	0.8704	0.9682	−0.0493	0.7724	0.5808	0.5423	0.2072	0.9882	0.1812	0.6954
6.00	0.9368	2.2039	0.9504	0.9724	−0.0559	0.8100	0.5767	0.5816	0.2107	0.9907	0.2061	0.7197
7.00	0.9303	2.5455	1.0044	0.9658	−0.0865	0.8539	0.5716	0.6094	0.2193	0.9904	0.2117	0.7590
8.00	0.9207	2.7232	1.0339	0.9598	−0.0124	0.8410	0.5854	0.6179	0.2279	0.9828	0.2273	0.7563

*Note:* The correlation coefficient between estimated and measured fMRI signal (*r*) and the linear fit with intercept *α* and slope *β* are presented for each dispersion coefficient and each operating frequency.

**FIGURE 5:**
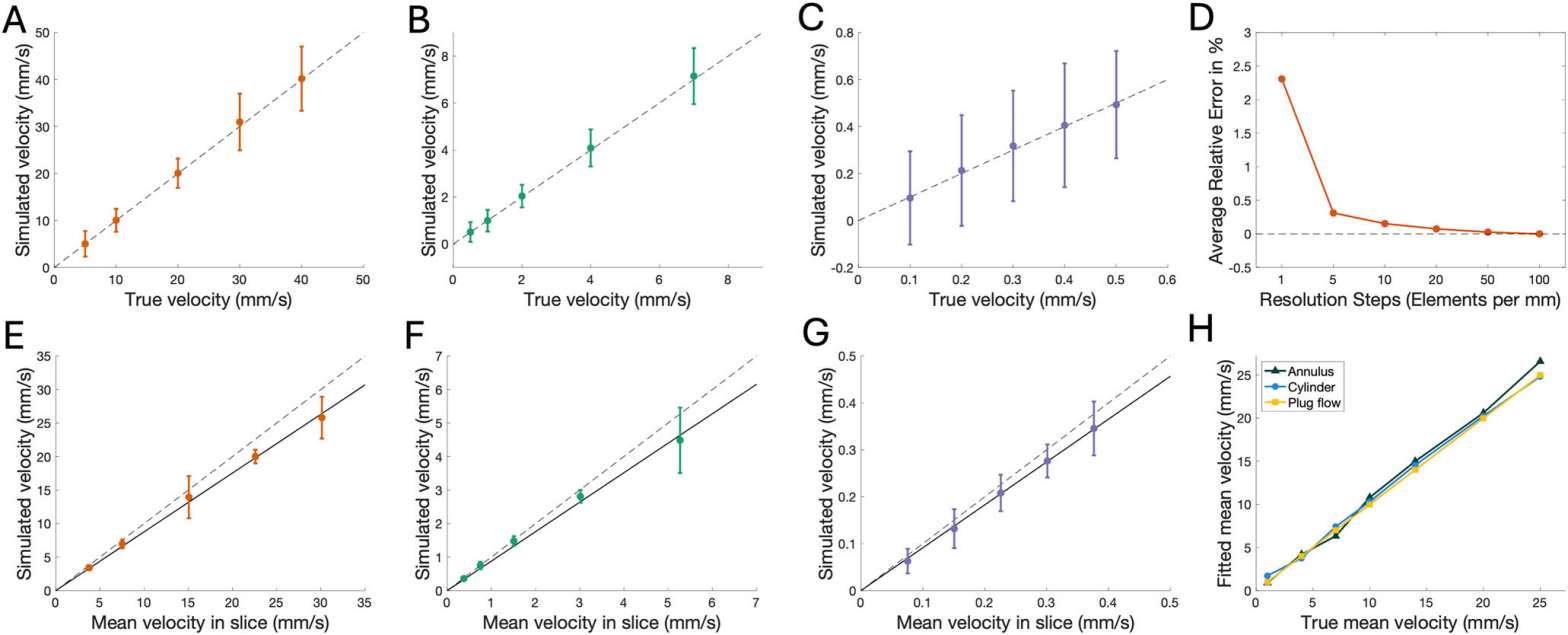
Digital phantom validation results assessing model robustness and assumptions. (A–C) Correlation between fitted and true velocities adding noise to the signal for the (A) cardiac, (B) respiratory, and (C) low‐frequency components. The error bars represent the 95% Reference Interval of the population. (D) Relative velocity error as a function of simulation spatial resolution (elements per mm). (E–G) Projected fitted velocities versus true velocities when approximating a simulated funnel geometry using straight‐pipe geometry for the (E) cardiac, (F) respiratory, and (G) low‐frequency components. The error bars represent the 95% Reference Interval of the population. (H) Mean velocities obtained by fitting a plug flow to simulated signals of different flow profiles plotted against the underlying mean velocities.

The digital phantom simulations demonstrated that the proposed model is highly robust to physiological noise, maintains high accuracy across varying spatial resolutions, and reliably estimates velocities even when simplifying complex geometries. Comprehensive performance metrics and correlation analyses for these computational validations across all frequency bands are detailed in Figure 5. The estimated mean velocities obtained by fitting a plug flow to the signals of different flow profiles showed strong agreement with the corresponding mean velocities of the underlying flow profiles. The correlation coefficients between the fitted and actual mean velocities were 0.999 for both laminar flow in a cylinder and laminar flow in an annulus. Per design, there was a perfect correlation with plug flow. A summary of these comparisons is presented in Figure 5H.

### 3.2 CSF Flow Rates and Volumes

**FIGURE 6:**
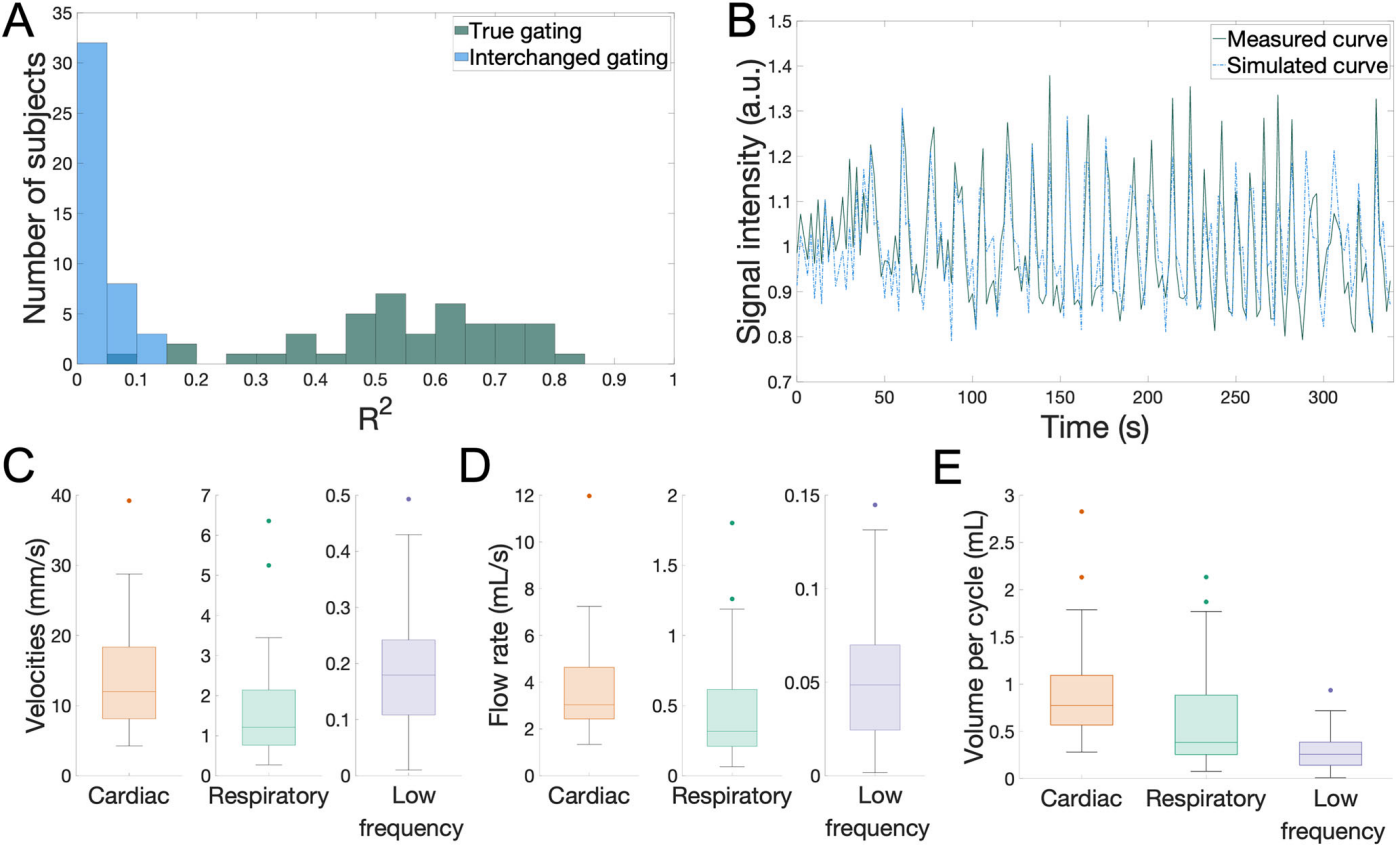
Results from the simulations run on the participants of this study (*N* = 43). (A) Correlation coefficients between simulated and measured CSF signal for each subject, both with each participant's true gating and interchanged gatings. (B) Example fit of the CSF signal. (C) Estimated velocities for each subject and physiological component. Note the different scales on the vertical axes. (D) Estimated flow rates for each subject and physiological component. Note the different scales on the vertical axes. (E) Estimated stroke volumes over each of the physiological cycles for each subject.

**FIGURE 7:**
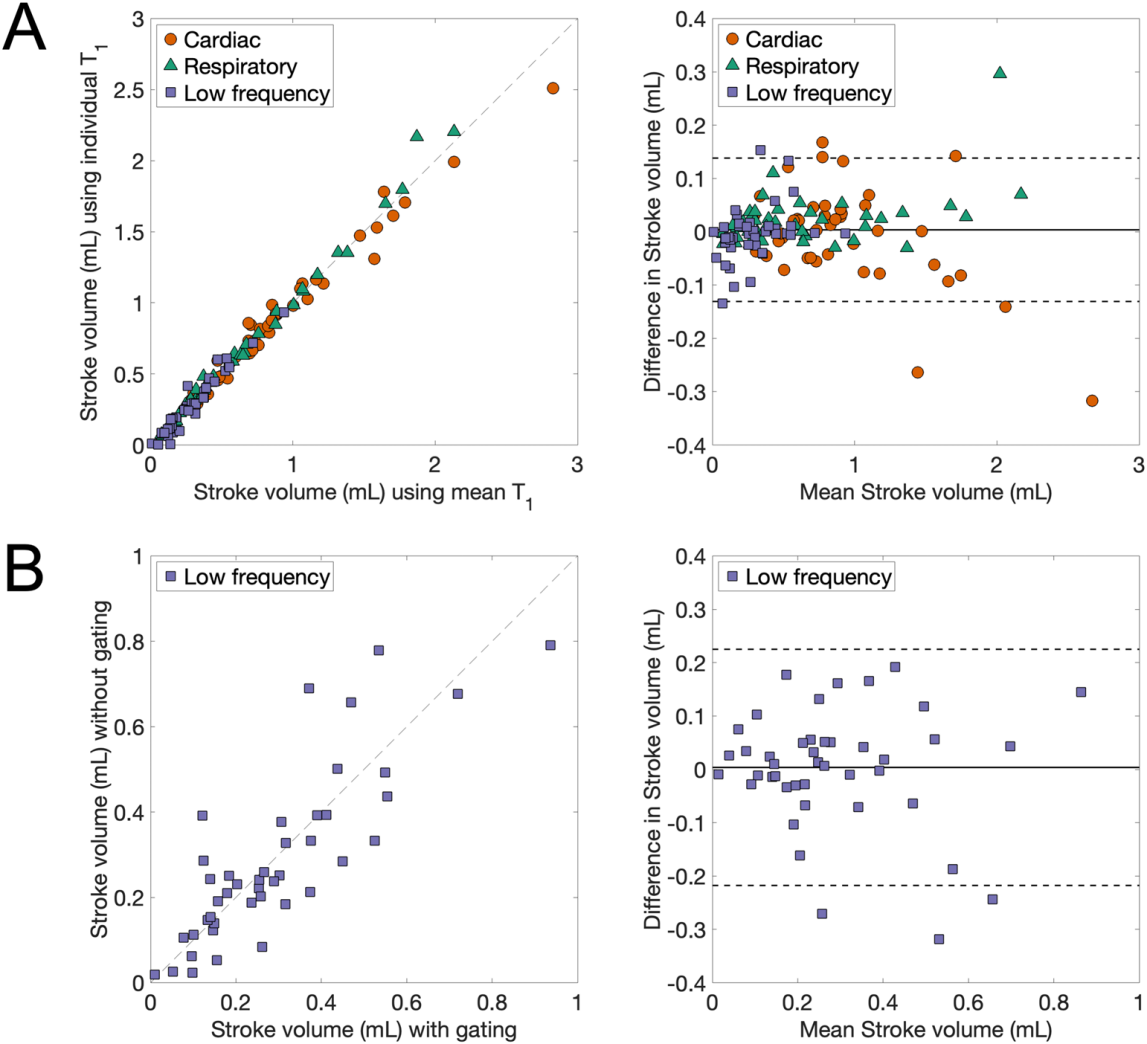
(A) Bland–Altman analysis of CSF stroke volumes estimated using the cohort‐mean T_1_ versus individual subject T_1_‐values. The difference in stroke volume is calculated as the estimate utilizing the individual T_1_ minus the estimate utilizing the cohort‐mean T_1_. The figure presents the results across the three physiological domains. (B) Bland–Altman analysis of low‐frequency CSF stroke volume estimates with and without external gating. The difference in stroke volume is calculated as the estimate with external gating minus the estimate without external gating.

When applied to a whole‐brain fMRI data set of *N* = 43 individuals (68–82 years, 19 women), quantitative estimates of the CSF flow at the cranio‐cervical junction could be obtained in all subjects. The simulated and measured CSF inflow displayed *R* ^2^ values of 0.57 [0.47, 0.67], supporting the appropriateness of the model; see Figure 6A for the distribution of individual *R* ^2^ values. Figure 6A also contains the outcome of a negative‐control test, where the inflow signal was deliberately matched to cardiac, respiratory, and BOLD signals of another subject, verifying that a good fit could not be obtained by chance. A typical fit is displayed in Figure 6B (*R* ^2^ = 0.64). The estimated velocity amplitudes of the cohort were 12.0 [8.1, 18.4] mm/s for the cardiac component, 1.21 [0.76, 2.14] mm/s for the respiratory component, and 0.18 [0.11, 0.24] mm/s for the low frequency component (Figure 6C). After factoring in the area of the CSF region, the corresponding flow rates and stroke volumes were calculated to be 3.04 [2.43, 4.63] mL/s and 0.77 [0.57, 1.09] mL for the cardiac cycle, 0.32 [0.21, 0.61] mL/s and 0.38 [0.26, 0.88] mL for the respiratory cycle, and 0.049 [0.024, 0.070] mL/s and 0.26 [0.14, 0.39] mL for the low frequency cycle (Figure 6D,E). Moreover, the agreements between the stroke volumes estimated using a fixed T_1_‐time versus the individually set T_1_‐times were high, as indicated by small systematic differences (1%, 3%, and 2% for cardiac, respiratory, and low frequency oscillations, respectively), and high correlation values (0.987, 0.996, and 0.971 for cardiac, respiratory, and low frequency oscillations, respectively) (see Figure 7A). In addition, the low frequency CSF stroke volumes displayed a high correlation between fits with and without cardiac/respiratory gating (*r* = 0.83), indicating the model's robustness (see Figure 7B).

## 4 Discussion

We propose a method to characterize cardiac, respiratory and low frequency velocity components of CSF motion compatible with a standard whole‐brain fMRI implementation, where CSF is captured in the bottom slice of the imaging volume. Using this method, we could show that CSF flow at the foramen magnum was dominated by cardiac related oscillations, with lower stroke‐volumes for the respiratory and low frequency oscillations. The method was inspired by recent advancements in utilizing the inflow‐related signal enhancements that develop because of CSF oscillations in and out of the imaging volume (e.g., at the fourth ventricle or at the foramen magnum) [14, 15, 16]. However, the approach presented here provides some major benefits compared to previous studies. Firstly, the method provides quantitative estimates of CSF velocities, based on a model that does not depend on specific imaging parameters and is verified with phantom experiments. Although qualitative assessments are useful for identifying population trends, they lack the quantitative precision required for individual comparisons. Since flow is a product of velocity and area, two subjects with similar signal intensities can have different flow rates due to variations in foramen magnum area. Furthermore, our results demonstrated that the method is robust using a typical rather than measured T_1_‐time of CSF, supporting use in datasets where the CSF T_1_‐acquisition is not available. Secondly, using recordings of cardiac and respiratory cycles, individual contributions from physiological cycles could be estimated. Because the TR in standard fMRI protocols typically exceeds the Nyquist limit for cardiac pulsations, such decompositions are generally not feasible directly from the raw single‐slice signal without specialized reconstruction techniques. The method presented in the current study is intended to offer means of retrospectively assessing CSF flow in large‐scale studies that included fMRI acquisitions where edge‐slice CSF is visible, as would be expected in studies employing whole‐brain fMRI. If physiological recordings are unavailable or of inconsistent quality across scanning sites, data‐driven signal processing techniques can extract these signals directly from raw fMRI data for datasets with short TR [42]. Otherwise, deep‐learning frameworks [43, 44] can provide robust estimates of these signals directly from the fMRI data by leveraging the predictable influence of systemic physiological cycles on the BOLD signal in multislice fMRI [45].

In the present study we applied the method to analyze CSF flow and stroke volumes at the cranio‐cervical junction, where we found median cardiac velocity amplitudes of 12.0 mm/s and respiratory velocity amplitudes of 1.21 mm/s. Previous literature has reported CSF velocities and stroke volumes in this region using PC‐MRI, with cardiac velocities by Yildiz et al. 2017 and 2022 to be 9.7 ± 3.3 mm/s [46] and 17.5 ± 4.0 mm/s [47] respectively, compared to Takizawa et al. that reported cardiac velocities of ∼10 ± 5 mm/s [48]. The corresponding respiratory velocities in the same studies were 5.8 ± 4.0 mm/s [46], 6.8 ± 3.4 mm/s [47] and ∼ 3 ± 1 mm/s [48]. However, in the study by Yildiz et al. 2022 they consider a single voxel approach that captures peak velocity, rather than the spatial average CSF velocity as we do. Wåhlin et al. have reported cardiac stroke volumes at cervical level to be 0.773 ± 0.231 mL [1] while Liu et al. presented values of ∼0.6 mL [49], both correspond very well with our estimate of 0.77 mL. The respiratory stroke volumes at cervical level during free breathing have been reported to be 0.5 ± 0.2 mL [50] and 0.33 ± 0.2 mL [51], both that are in good agreement to our estimations (median 0.38 mL). Moreover, a study using guided breathing reported respiratory stroke volumes of 0.99 mL (range: 0.54–1.66 mL) [52]. These values exceed our free‐breathing estimations, likely because breathing mode influences CSF modulation [53], where guided breathing tends to amplify respiratory effects compared to spontaneous breathing. Importantly, these effects may vary with age [54], and since the participants in our cohort were older than those in the studies used for comparison, some caution in the comparisons of absolute values is warranted.

For the low frequency component of the CSF velocity at foramen magnum, no established reference values exist. However, a crude estimation of CSF velocity can be derived from intracranial pressure (ICP) recordings when analyzing slow waves variations of ICP. The total change in intracranial volume (∆V) is governed by the definition of intracranial compliance (C) and the amplitude of the pressure changes (∆P), such that ∆V=C·∆P [55]. The specific volume of CSF displaced into the spinal canal ($∆V_{\text{spinal}}$) is then determined by scaling this total volume by the spinal fraction of the compliance ($r_{\text{spinal}}$), yielding $∆V_{\text{spinal}}=∆V\cdot r_{\text{spinal}}$. Assuming the slow wave variations exhibit sinusoidal behavior with an operating frequency f, the peak volumetric flow rate is the time derivative of the displaced volume (2$\pi f\cdot ∆V_{\text{spinal}}$). The corresponding velocity amplitude is obtained by dividing this flow rate by the cross‐sectional area of the CSF flow pathway at the foramen magnum (A), resulting in the final expression $v=2\pi f\cdot ∆P\cdot C\cdot r_{\text{spinal}}/A$. For this calculation we used f=0.05 Hz, ∆P=0.8 mmHg [56], C=1.15 mL/mmHg [57], $r_{\text{spinal}}=35\%$ [4], and A=3.6 cm^2^ [58]. By combining these reference values, the estimated low frequency CSF velocity is approximately 0.28 mm/s. This velocity is of the same order of magnitude as observed in the present study. Moreover, the upper limit of the strong linear relationship observed for the low frequency component in the phantom measurements is approximately five times the estimated velocity, indicating that our method performs reliably within the expected physiological range.

Previous studies have presented an approach to utilize the inflow effect to relate CSF flow and brain activity by calculating the correlation between the CSF signal and global BOLD signal. This method has been successful in linking a reduced coupling between CSF flow and brain activity to neurodegenerative diseases and cognitive decline [15, 16]. Nevertheless, these results may not fully capture the magnitude or change of the underlying CSF flow, as the correlation measure only reflects degree of temporal coupling. An analytical model to estimate CSF flow from the inflow signal has been used, where the signal equations derived by Gao et al. [25] are utilized to translate the fMRI signal into flow rates [59]. However, a limitation of that approach is its inability to capture the oscillatory nature of CSF flow. Because the signal equations are derived using simplified assumptions, such as unidirectional flow and single‐slice excitation, such a model is more suitable for blood flow estimations [24, 26]. Another approach is to use PC‐MRI and BOLD fMRI in an interleaved fashion [60], which can capture both flow measurements and the BOLD signal accurately. Such specialized imaging may however not always be feasible (e.g., in ongoing longitudinal aging and dementia studies), whereby a method to retain similar information from standard imaging is desirable. Moreover, a recently presented approach based on multiple slice excitation together with artificial intelligence has shown promising results to translate the inflow signal to velocities when using fMRI data with short repetition times [61]. Conceptually, our modeling approach shares similarities with the methodology presented by van der Voort et al. [62], particularly in the use of peripheral physiological recordings (cardiac and respiratory) to isolate high‐frequency CSF dynamics from sequences sampled below the Nyquist limit. However, the fundamental mechanics of the two approaches diverge significantly. Although van der Voort et al. decompose quantitative displacement data acquired from dedicated motion‐sensitive sequences, our framework utilizes a Bloch‐simulation approach to translate the non‐quantitative, inflow‐related signal fluctuations of standard fMRI into quantitative flow rates.

### 4.1 Limitations

Although periodic in‐ and outflow of CSF cause signal variations and systematic spin‐history effects [60] that our forward model is explicitly designed to resolve, subject motion introduces unpredictable, non‐oscillatory spin‐history changes that violate the modeled fluid dynamics and remain exceedingly difficult to correct retrospectively [63]. Because of this, we have excluded participants with high movement during the scanning since they are expected to yield unreliable results for this kind of analysis. The fMRI acquisition is also sensitive to B_0_ fluctuations which may mainly be affected by respiration. However, the respiratory B_0_ fluctuations at cervical level 1 and the brain have been shown to be relatively small [64]. Moreover, the model assumes the CSF velocity distribution to be well approximated by a plug flow (i.e., only a spatial average of the velocity is calculated), although the underlying velocity distribution is unknown. However, as simulations showed that the fitted velocities of plug flow display a high correlation to the underlying mean velocities for various flow profiles, the impact of this shortcoming is probably limited. Furthermore, given that this method is intended for the retrospective analysis of fMRI cohorts, the bottom slice may not always be perfectly orthogonal to the foramen magnum. However, based on the principle of mass conservation, any slice obliquity is expected to increase the cross‐sectional area, effectively compensating for the decrease in the estimated velocity component. Consequently, the calculated volumetric flow rate theoretically remains unaffected in such cases. Additionally, Equation (1) does not include a term for the net flow of CSF. Previous research suggests that the CSF production rate in humans is approximately 0.5 mL/min [65]. If 50% of the produced CSF is assumed to pass through foramen magnum [66], with an area of 3.6 cm^2^ [58], the resulting net velocity would be about 0.01 mm/s. Since this net velocity is substantially lower than the oscillatory velocities, it was considered negligible for the model and therefore disregarded in this study to keep the model complexity down. Moreover, the fMRI data were acquired after the intravenous injection of gadolinium, which affects T_1_ relaxation. However, our inclusion of pre‐ and post‐fMRI T_1_‐mapping ensured accurate T_1_ values for the corresponding timepoints. Lastly, the model was only validated using one set of imaging parameters representative of typical large‐scale fMRI‐datasets. However, we see no intrinsic limitation in the approach that would render the method infeasible for variations in parameter settings (e.g., TR, slice‐thickness, slice order, between‐slice acceleration). Furthermore, in the phantom validations, velocity estimations for the low frequency oscillations broke down at non‐physiological high velocities (> ∼1.5 mm/s). We tested this extreme range due to a lack of established literature values. However, the failure point lies well outside expected in vivo conditions. Because this breakdown did not occur in the digital simulations, we suspect it is an artifact specific to the experimental phantom setup, potentially introducing unexpected flow profile deviations or saturation limits that are not present in the idealized model.

## 5 Conclusions

By modeling spin‐history we were able to quantify CSF flow from the inflow effect in fMRI data. The cardiac, respiratory and low frequency flow rates could be separated and cyclic volume variations (i.e., stroke volume) associated with each cycle could be calculated. CSF motion was dominated by the cardiac cycle since this cycle was faster and of greater stroke‐volume compared to the other two cycles. Importantly, the proposed framework requires no specialized pulse sequence. It was designed to be flexible and readily adaptable to standard fMRI acquisitions, provided that physiological signals are available along with edge‐slice CSF coverage.

## Funding

This work was supported by Hjärt‐Lungfonden (20210653), Vetenskapsrådet (2021‐00711_VR/JPND, 2022‐04263), Stiftelsen för Strategisk Forskning (RMX18‐0152).

## Supporting information


**Figure S1:** (A) Histograms of scores along the first principal component (PC1) of the voxel time series for a representative subject (median Silhouette score), used here for visualization of class separability. The bimodal distribution reflects two distinct voxel populations corresponding to CSF and non‐CSF tissue. (B) Pooled PC1 density across all 48 subjects, aligned to individual decision boundaries (centered at 0). The deep valley at the boundary and two distinct peaks confirm that the two classes remain consistently separable across the cohort. (C) Cluster validation using Silhouette and Elbow metrics. The Silhouette Coefficient peaks at 0.45 for two clusters. The Elbow Method shows that the second cluster explains 24% additional variance, whereas a third cluster provides only marginal improvement (12%), supporting the selection of a binary model.
**Figure S2:** Schematic illustration of the phantom setup. The validation was performed using a hollow cylinder that mimics the oscillatory flow of cerebrospinal fluid. A syringe pump created a sinusoidal flow through the cylinder, while a second passive syringe on the opposite end moved in sync with the fluid displacement. The cylinder was cast in agar to avoid interference between the water and the container walls. The fMRI volume was centered to capture the middle of the cylinder in the bottom slice.

**Video S1:** Illustration of the simulation framework. CSF elements are initialized in an equispaced grid and assigned a starting magnetization. Their positions are updated continuously according to the prescribed velocity profile, with magnetization levels evolving based on specific fMRI sequence parameters and the Bloch equations. For visual clarity, this illustration utilizes a reduced number of elements compared to the full‐scale simulation.

## Data Availability

The data that support the findings of this study are available on request from the corresponding author. The data are not publicly available due to privacy or ethical restrictions.
