# Supplementary material for: Quantifying Cardiac, Respiratory, and Low Frequency Components of CSF Motion From fMRI Inflow Effects
Source: Magn Reson Med. 2026 May 17;96(4):1916–28. doi: 10.1002/mrm.70438 (PMC13418971; doi:10.1002/mrm.70438)
Supplement: Supplementary file 1 — Figure S1: (A) Histograms of scores along the first principal component (PC1) of the voxel time series for a representative subject (median Silhouette score), used here for visualization of class separability. The bimodal distribution reflects two distinct voxel populations corresponding to CSF and non‐CSF tissue. (B) Pooled PC1 density across all 48 subjects, aligned to individual decision boundaries (centered at 0). The deep valley at the boundary and two distinct peaks confirm that the two classes remain consistently separable across the cohort. (C) Cluster validation using Silhouette and Elbow metrics. The Silhouette Coefficient peaks at 0.45 for two clusters. The Elbow Method shows that the second cluster explains 24% additional variance, whereas a third cluster provides only marginal improvement (12%), supporting the selection of a binary model. Figure S2: Schematic illustration of the phantom setup. The validation was performed using a hollow cylinder that mimics the oscillatory flow of cerebrospinal fluid. A syringe pump created a sinusoidal flow through the cylinder, while a second passive syringe on the opposite end moved in sync with the fluid displacement. The cylinder was cast in agar to avoid interference between the water and the container walls. The fMRI volume was centered to capture the middle of the cylinder in the bottom slice. [file MRM-96-1916-s001.docx]

**Supporting Information**

**
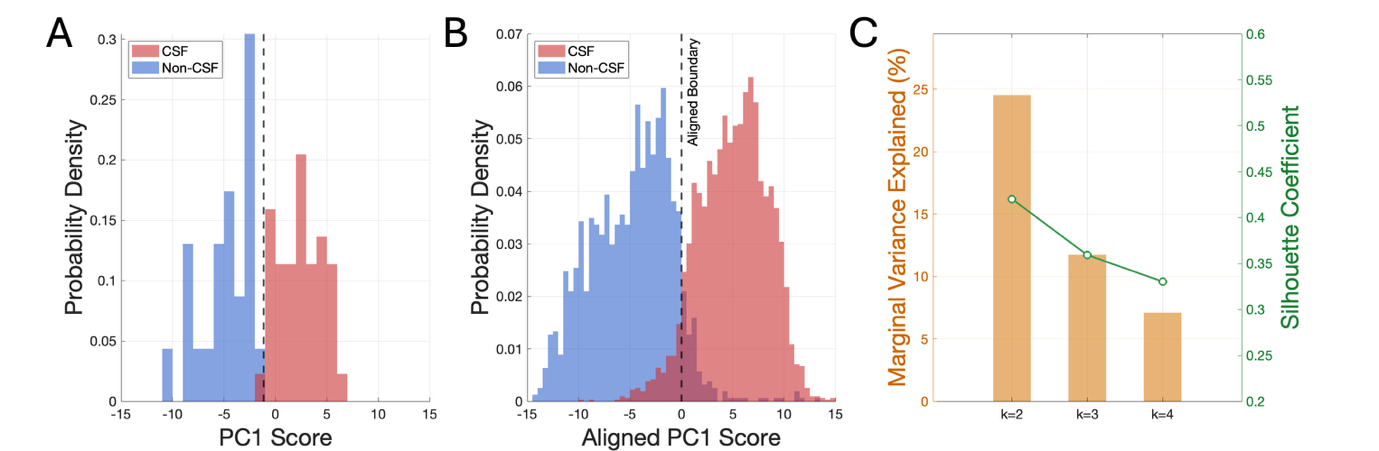
**

**Figure S1. (A)**Histograms of scores along the first principal component (PC1) of the voxel time series for a representative subject (median Silhouette score), used here for visualization of class separability. The bimodal distribution reflects two distinct voxel populations corresponding to CSF and non-CSF tissue. **(B)**Pooled PC1 density across all 48 subjects, aligned to individual decision boundaries (centered at 0). The deep valley at the boundary and two distinct peaks confirm that the two classes remain consistently separable across the cohort. **(C)** Cluster validation using Silhouette and Elbow metrics. The Silhouette Coefficient peaks at 0.45 for two clusters. The Elbow Method shows that the second cluster explains 24% additional variance, while a third cluster provides only marginal improvement (12%), supporting the selection of a binary model.


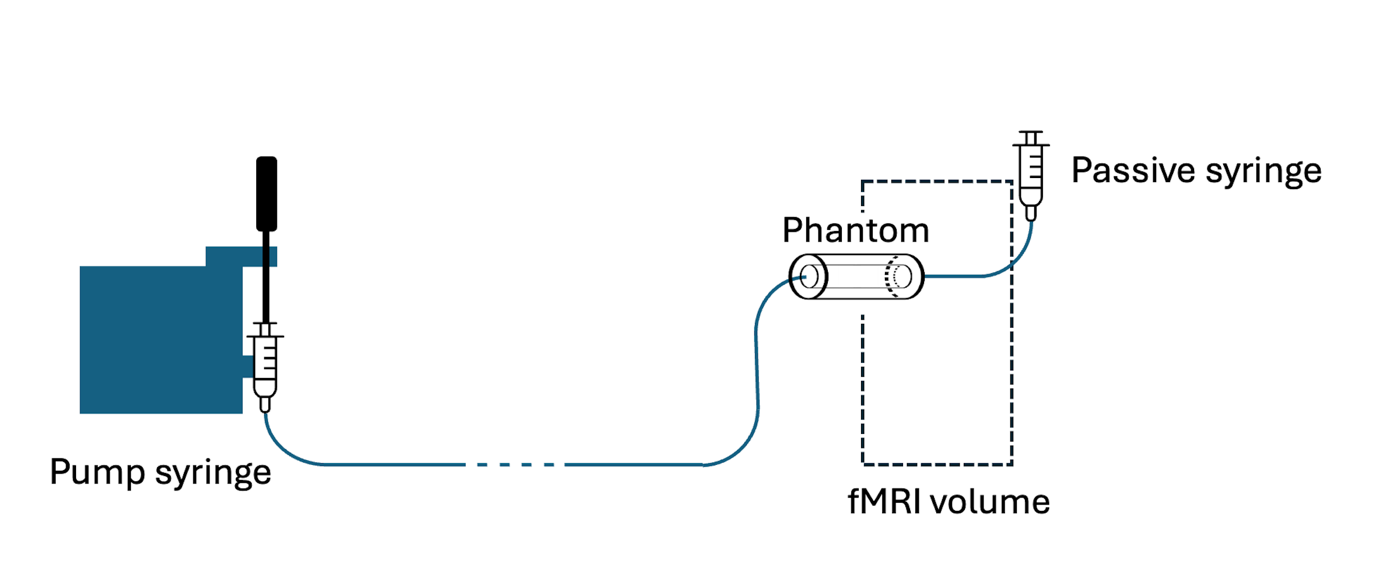


**Figure S2.** Schematic illustration of the phantom setup. The validation was performed using a hollow cylinder that mimics the oscillatory flow of cerebrospinal fluid. A syringe pump created a sinusoidal flow through the cylinder, while a second passive syringe on the opposite end moved in sync with the fluid displacement. The cylinder was cast in agar to avoid interference between the water and the container walls. The fMRI volume was centered to capture the middle of the cylinder in the bottom slice.

**Video S1.** Illustration of the simulation framework. CSF elements are initialized in an equispaced grid and assigned a starting magnetization. Their positions are updated continuously according to the prescribed velocity profile, with magnetization levels evolving based on specific fMRI sequence parameters and the Bloch equations. **Note:** For visual clarity, this illustration utilizes a reduced number of elements compared to the full-scale simulation.
